# High Cefuroxime Concentrations and Long Elimination in an Orthopaedic Surgical Deadspace—A Microdialysis Porcine Study

**DOI:** 10.3390/antibiotics11020208

**Published:** 2022-02-07

**Authors:** Sara Kousgaard Tøstesen, Maiken Stilling, Pelle Hanberg, Theis Muncholm Thillemann, Thomas Falstie-Jensen, Mikkel Tøttrup, Martin Knudsen, Emil Toft Petersen, Mats Bue

**Affiliations:** 1 Aarhus Denmark Microdialysis Research (ADMIRE), Orthopaedic Research Laboratory, Aarhus University Hospital, 8200 Aarhus N, Denmark; maiken.stilling@clin.au.dk (M.S.); pellehanberg@clin.au.dk (P.H.); matsbue@clin.au.dk (M.B.); 2 Department of Orthopaedic Surgery, Aarhus University Hospital, 8200 Aarhus N, Denmark; theithil@rm.dk (T.M.T.); thomfals@rm.dk (T.F.-J.); 3 Department of Clinical Medicine, Aarhus University, 8200 Aarhus N, Denmark; martinknudsen@clin.au.dk (M.K.); emiltp@clin.au.dk (E.T.P.); 4 AutoRSA Research Group, Orthopaedic Research Laboratory, Aarhus University Hospital, 8200 Aarhus N, Denmark; 5 Department of Orthopaedic Surgery, Aalborg University Hospital, 9640 Farsoe, Denmark; mikkel.toettrup@rn.dk

**Keywords:** microdialysis, periprosthetic joint infection, antibiotic prophylaxis, orthopaedic surgical deadspace, cefuroxime

## Abstract

Deadspace is the tissue and bony defect in a surgical wound after closure. This space is presumably poorly perfused favouring bacterial proliferation and biofilm formation. In arthroplasty surgery, an obligate deadspace surrounding the prosthesis is introduced and deadspace management, in combination with obtaining therapeutic prophylactic antibiotic concentrations, is important for limiting the risk of acquiring a periprosthetic joint infection (PJI). This study aimed to investigate cefuroxime distribution to an orthopaedic surgical deadspace in comparison with plasma and bone concentrations during two dosing intervals (8 h × 2). In a setup imitating shoulder arthroplasty surgery, but without insertion of a prosthesis, microdialysis catheters were placed for cefuroxime sampling in a deadspace in the glenohumeral joint and in cancellous bone of the scapular neck in eighteen pigs. Blood samples were collected as a reference. Cefuroxime was administered according to weight (20 mg/kg). The primary endpoint was time above the cefuroxime minimal inhibitory concentration of the free fraction of cefuroxime for *Staphylococcus aureus* (*f*T > MIC (4 μg/mL)). During the two dosing intervals, mean *f*T > MIC (4 μg/mL) was significantly longer in deadspace (605 min) compared with plasma (284 min) and bone (334 min). For deadspace, the mean time to reach 4 μg/mL was prolonged from the first dosing interval (8 min) to the second dosing interval (21 min), while the peak drug concentration was lower and half-life was longer in the second dosing interval. In conclusion, weight-adjusted cefuroxime *f*T > MIC (4 μg/mL) and elimination from the deadspace was longer in comparison to plasma and bone. Our results suggest a deadspace consolidation and a longer diffusions distance, resulting in a low cefuroxime turn-over. Based on theoretical targets, cefuroxime appears to be an appropriate prophylactic drug for the prevention of PJI.

## 1. Introduction

Arthroplasty surgery introduces an obligate deadspace surrounding the prosthesis. A deadspace is defined as the residual tissue void after soft tissue loss and/or bone removal with presumably poor perfusion. The deadspace volume is dependent on the type of prosthesis and surgical technique [1,2]. Following surgical closure, a haematoma is formed in the deadspace providing an environment with low oxygen tension and pH suitable for bacteria proliferation and biofilm formation [3]. Therefore, proper deadspace management is considered important to reduce the risk of developing a periprosthetic joint infection (PJI) [4]. PJI is a serious complication following arthroplasty surgery associated with significant morbidity for the patient [5]. In spite of great advancements in the field of orthopaedic devices and surgical techniques, the introduction of foreign materials is still associated with a substantial risk of infection due to increasing bacterial virulence [6,7]. *Staphylococcus aureus* (*S. aureus*) remains the most causative aetiology in PJIs [8,9,10]. To protect the prosthesis surface from bacterial colonisation and ensure host integration, it is paramount to achieve adequate perioperative prophylactic antibiotic concentrations in the deadspace surrounding the prosthesis [11,12].

To dynamically assess target antibiotic deadspace concentrations, microdialysis is a favourable method and has previously been employed for sampling of various antibiotics in different solid tissues [13,14,15]. Microdialysis allows for sampling of the free and active antibiotic concentrations simultaneously from multiple target tissues.

Cefuroxime is commonly utilised as a prophylactic antibiotic agent in arthroplasty surgery due to its broad-spectrum effect against Gram-positive and Gram-negative bacteria. Cefuroxime exhibits time-dependent bacterial killing, and time above the minimal inhibitory concentration of the free unbound and active fraction (*f*T > MIC) of cefuroxime is considered the best predictor of therapeutic and prophylactic efficacy [16,17]. For cefuroxime, the *S. aureus* planktonic epidemiological cut-off value (ECOFF) is 4 µg/mL [18].

This study aimed to employ microdialysis in a porcine model to investigate weight-adjusted cefuroxime *f*T > MIC (4 μg/mL) in an orthopaedic surgical deadspace in comparison with concurrent plasma and bone values during two dosing intervals (8 h × 2).

## 2. Results

All pigs completed the study and all dialysates were obtained from all catheters. Mean relative recovery (SD) was 62% (15) in deadspace and 34% (10) in bone. For one bone catheter, relative recovery could not be determined. Since the dialysate concentrations from this catheter resembled those of the other bone catheters, the mean value of the remaining bone relative recoveries was applied for this catheter.

### 2.1. Comparison of Weight Groups

When comparing the humeral head size across the weight groups, there was a positive correlation between pig weight and the humeral head size (Table 1). Nonetheless, deadspace *f*T > MIC (4 μg/mL) and deadspace penetration were comparable between weight groups (Table 1). Since *f*T > MIC (4 μg/mL) was the main target, data from all 18 pigs (three groups) were pooled as one study group for further description.

### 2.2. fT > MIC (4 μg/mL) and Time to Reach A Mean Concentration of 4 μg/mL

**Figure 1:**
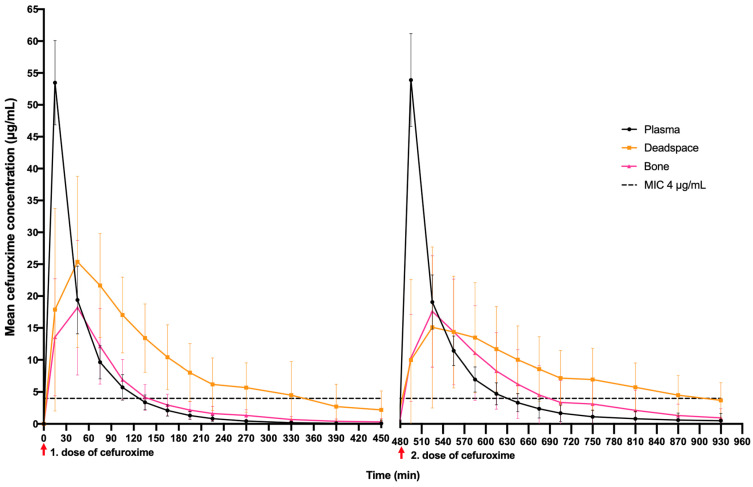
Mean concentration–time profiles across weight groups of cefuroxime in plasma, deadspace and bone over two dosing intervals. SD visualised with bars.

Mean concentration/time profiles for all 18 pigs are presented in Figure 1. During the two dosing intervals (960 min), the deadspace mean *f*T > MIC (4 μg/mL) (95% CI) was 605 min (552–658) and longer compared to plasma, 284 min (232–337), and bone, 334 min (282–387). Furthermore, the mean deadspace *f*T > MIC (4 μg/mL) was longer in the second dosing interval (338 min, 95% CI: 284–391) compared to the first dosing interval (277 min, 95% CI: 224–331) (Table 2). Corresponding *f*T > MIC (4 μg/mL) results in the percentage of the two dosing intervals are depicted in Table 2. Within 10 min of the first dosing interval, a mean concentration of 4 μg/mL was reached in all the investigated compartments. In the second dosing interval, only deadspace presented a longer time to reach a mean concentration of 4 μg/mL in comparison to the first dosing interval (21 min) (Table 3).

### 2.3. Pharmacokinetic Parameters

All plasma and bone pharmacokinetic parameters were comparable between the first and second dosing interval. While deadspace cefuroxime exposure (described by AUC) were comparable between the first and second dosing interval, deadspace C_max_ was significantly lower in the second dosing interval (19 µg/mL) compared to the first dosing interval (27 µg/mL), and T_max_ and T_½_ were significantly longer in the second dosing interval (89 and 407 min, respectively) compared with the first dosing interval (57 and 151 min, respectively). Although plasma C_max_ were higher than deadspace C_max_ in both dosing intervals, deadspace presented with a significantly higher AUC in comparison to plasma in both dosing intervals (Table 4).

## 3. Discussion

This study investigated weight-adjusted cefuroxime *f*T > MIC (4 μg/mL) in a relevant orthopaedic deadspace during two dosing intervals (8 h × 2). The main finding was a significantly longer mean *f*T > MIC (%*f*T > MIC) for the *S. aureus* ECOFF MIC (4 μg/mL) in deadspace of 605 min (67%), compared with plasma, 284 min (32%), and bone, 334 min (37%), during the two dosing intervals. A mean cefuroxime concentration of 4 µg/mL was reached within 10 min in all compartments during the first dosing interval. In the second dosing interval, only deadspace presented a longer time to reach a concentration of 4 µg/mL (21 min) in comparison to the first dosing interval. The bone penetration ratios achieved in this study were comparable to both clinical and porcine studies [19,20], while deadspace penetration ratios have not previously been examined. Our results may support the curious notion of impaired perfusion and deadspace filling with haematoma after wound closure, summarised with the following key findings:

(1) Deadspace *f*T > MIC (4 µg/mL) was longer than that of both bone and plasma, especially in the second dosing interval.
(2) Plasma C_max_ was twice as high as deadspace C_max_, but deadspace AUC was significantly higher than plasma AUC.
(3) Deadspace penetration was higher than that of bone in both dosing intervals.
(4) In the second dosing interval, deadspace penetration was significantly delayed compared to the first dosing interval, and a lower mean concentration was found.
(5) Deadspace elimination was significantly longer in the second dosing interval compared with first dosing interval, and more than twice that of plasma and bone in both dosing intervals.

A deadspace is formed by tissue fluid and bleeding immediately following surgery. As the deadspace is gradually filled and consolidates, the cefuroxime contribution from bleeding is reduced and the penetration (and elimination) of cefuroxime becomes more dependent on diffusion, which, in turn, may also deteriorate due to consolidation of the haematoma. Thus, it seems theoretically relevant to select an antibiotic agent with time-dependent bacterial killing (e.g., cefuroxime), rather than concentration-dependent killing, for perioperative antibiotic prophylaxis in arthroplasty surgery. However, the presented low deadspace turnover with a potential decreasing cefuroxime penetration over time may result in low and sub-therapeutic concentrations in following dosing intervals. Therefore, it also seems important to reach high cefuroxime concentrations at wound closure to load the deadspace and protect against infection development. Assuming that the elimination rate is dose independent, even a small increase in the cefuroxime dose and repeated cefuroxime administration at wound closure may improve deadspace *f*T > MIC (4 μg/mL) significantly in the following dosing intervals. However, any potential effect or clinical need for continued postoperative prophylaxis cannot be evaluated with the present setup and necessitates further studies.

It is important to appreciate that the definition of adequate target *f*T > MIC for prophylactic cefuroxime treatment in arthroplasty surgery is not well established. However, for perioperative antibiotic prophylaxis, it is generally recommended that target tissue concentrations, as a minimum, exceed relevant MIC from incision and until wound closure [16,21]. Prudently, assessment of *f*T > MIC efficacy depends on the MIC evaluated, and will depend on local susceptibility patterns and different strains of bacteria. Based on *S. aureus* theoretical targets, our results suggest that a weight-adjusted dose of 20 mg/kg cefuroxime, administered no closer than 10 min prior to surgery, will provide therapeutical concentrations for a mean time of 277 min in deadspace and 130 min in bone, which will suffice for most arthroplasty surgeries. For surgeries of longer duration, simple measures can be applied to ensure prolonged *f*T > MIC (4 μg/mL) in all relevant tissues, e.g., repetitive dosing, with administration of the second cefuroxime dose after approximately 2 h, or continuous infusion [20,22]. As for postoperative prophylaxis, it is still widely debated as to how long the prophylactic antibiotic treatment should be continued after wound closure in arthroplasty surgery [23,24].

Surgical and pharmaceutical deadspace management is important to protect the prosthesis from colonisation [4,6]. Although weight-adjusted cefuroxime dosing may provide theoretically adequate prophylactic concentrations, a large and poorly perfused deadspace with a long diffusion distance may inhibit the ability of the immune system to penetrate to the deadspace surrounding the prosthesis [2]. Moreover, the surgical trauma may create conditions in which the body’s immune defence is compromised [25]. Abreast of increasing bacterial virulence when introducing foreign materials [26,27], and the ability of bacteria to develop a self-protective biofilm on the implant surface [28] as early as within the first 24 h after surgery [29], the interstitial environment surrounding the prosthesis is also often referred to as a vulnerable region with local immune depression [30]. Thus, taking all of these factors into account, the role of adequate prophylactic antibiotic administration may be particularly important to protect the prosthesis from colonisation, and may require antibiotic concentrations several times higher than the employed planktonic targets in arthroplasty surgery [31].

Some limitations should be mentioned. The applied internal calibrator, meropenem, has only demonstrated stability in room temperature for up to 12 h, whereas we sampled over 16 h [32,33]. Thus, a potential degradation of the internal standard in the end of the sampling interval cannot be excluded, which could lead to falsely higher relative recovery values with corresponding falsely lower cefuroxime concentrations. In terms of the physiology and anatomy of the model, pigs and humans are comparable in several areas [34]. However, as this study was performed on healthy juvenile pigs (aged 6 months), important interspecies differences regarding comorbidity or age-related changes could not be evaluated. Moreover, studies have demonstrated that pigs tend to have a faster cefuroxime elimination (shorter *f*T > MIC and T_½_) in comparison to humans in comparable experimental and clinical designs [20,35]. Thus, the prolonged cefuroxime deadspace elimination and *f*T > MIC presented in this study may be even more prolonged in clinical practice. For future studies, insertion of a prosthesis to mimic a true arthroplasty setup (with/without cement) and proximate periprosthetic evaluation of cefuroxime concentrations should be considered, since the presence of a prosthesis might affect local perfusion conditions and limit the deadspace. Further, it should be recognised that the surgical procedure and placement of catheters was performed before cefuroxime administration in contrast to a clinical setting, where cefuroxime is given preoperatively, and thus before tissue trauma and consolidation of a haematoma. Although our surgical procedure imitates a true perioperative situation, preoperative cefuroxime administration may influence the initial accumulation of cefuroxime in the deadspace haematoma affecting deadspace *f*T > MIC and penetration. It could be interesting to extend the sampling period to evaluate if deadspace penetration is continuously delayed and decreased in the following dosing intervals or whether/when the haematoma dissolves. Lastly, the applied descriptive statistics are restricted to the actual data. Along with the half-life (60–90 min) of cefuroxime, short infusion time and initial sampling interval of 30 min, this may explain the variability of the first samples in particular in each of the investigated compartments. A future model-based approach may be considered to more sufficiently accommodate the variations and predict the effect of, e.g., other dosing regimens.

## 4. Materials and Methods

The study was approved by the Danish Animal Experiments Inspectorate (License No. 2017/ 15-0201-01184), complying with Danish laws regulating experimental animal research. This study was conducted at the Institute of Clinical Medicine, Aarhus University Hospital, Denmark. Laboratory analyses were utilised at the Department of Clinical Biochemistry, Lillebaelt Hospital Vejle, Denmark. Adhering to recommendations of Replacement, Reduction or Refinement in animal studies [36], the same 18 pigs were included in a study investigating the effect of weight-based cefuroxime dosing on orthopaedic relevant target tissue concentrations in the first dosing interval [37].

### 4.1. Study Design and Anaesthetic

Eighteen female pigs (Danish Landrace) were included into three weight groups; 53–57, 73–77, and 93–97 kg, with six pigs in each group. The pigs were anaesthetised, combining continuous infusion of propofol (450–750 mg/h) and fentanyl (0.6–0.85 mg/hour) during surgery and the sampling period. Arterial blood pH was monitored and regulated through ventilation and kept within the range of 7.42 to 7.54. Core temperature was kept within the range of 35.6 to 38.6 °C, regulated with blankets or ice bags. Glucose was substituted when needed and 0.9% NaCl was given continuously (150 mL/h) to maintain normohydration. At the end of the sampling period, the pigs were euthanised with an intravenous injection of pentobarbital.

### 4.2. Surgical Procedure

**Figure 2:**
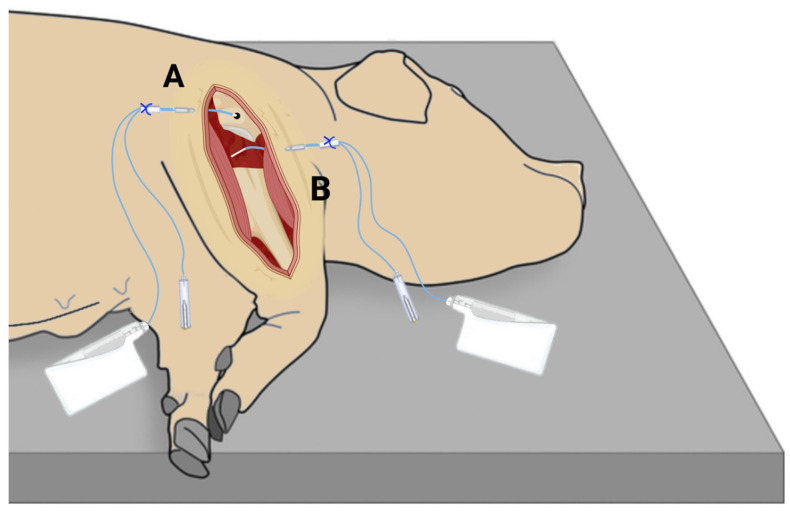
Illustration of the placement of microdialysis catheters. (**A**) cancellous bone of scapular neck; (**B**) deadspace. Figure 2 was created with BioRender.com [42].

With the pigs positioned in left lateral recumbency, the surgical intervention performed in this study imitated a shoulder arthroplasty surgery creating a deadspace in the glenohumeral joint, but without insertion of a prosthesis. To reach the glenohumeral joint of the right shoulder, a lateral skin incision exposed the underlying musculature and the infraspinatus muscle was released from the humeral attachment. A deadspace was created by excision of the humeral head using an oscillating saw. A microdialysis catheter (membrane length: 30 mm) was placed in the deadspace by passing a splitable introducer through the supraspinatus muscle and into the deadspace of the glenohumeral joint. A catheter (membrane length: 10 mm) was placed in a drill hole (ø: 2 mm, depth 20–25 mm) in the cancellous bone of the scapular neck, approximately 10 mm above the superior part of the glenoid labrum (Figure 2). The microdialysis catheters were fixed to the skin with sutures to avoid displacement. Hereafter, perfusion with 0.9% NaCl containing 5 μg/mL meropenem allowed individual calibration of the microdialysis catheters using the internal standard method [38]. Thirty minutes of tissue equilibration was allowed for.

**Figure 3:**
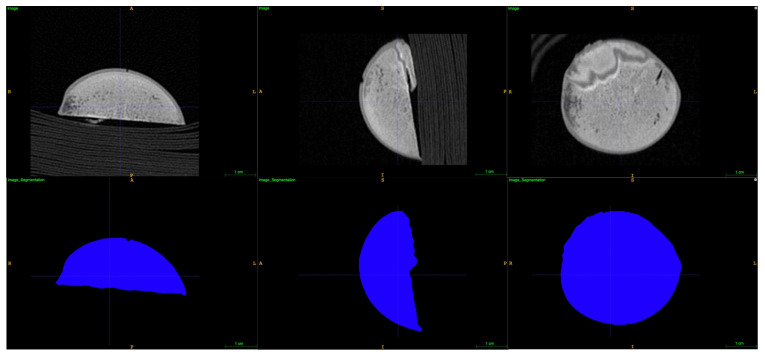
Illustration of computed tomography (CT) scans and segmentations used to determine volume of the excised humeral head.

A Siemens scanner (SOMATOM Definition Flash; Siemens Healthcare, Erlangen, Germany) was used to perform post mortem computed tomography (CT) of the excised humeral head, estimating its volume, and thus an indirect indicator of the deadspace volume (Figure 3). Estimates of CT volumes were obtained with the following settings: peak voltage of 120 kVp and exposure of 168 mAs, slice thickness of 0.6 mm and pixel spacing of 0.11 × 0.11 mm. From the CT scan, 3D surface models of the humeral head were generated using a fully automated graph-cut segmentation method [39,40,41]. A custom-written Python (Python.org, version 3.6.6) program utilising Virtualization Toolkit (Kitware, version 8.1.0) was applied to determine the volume of the humeral head in mm^3^.

### 4.3. Cefuroxime Administration, Sampling and Handling of Samples

The overall sampling time was 16 h, divided by two dosing intervals of 8 h. At time 0 min, cefuroxime was administered intravenously as a weight-adjusted bolus (20 mg/kg) over 10 min. From 0–240 min, dialysates were collected every 30 min and from 240–480 min, every 60 min. Blood samples were collected from a central venous catheter as a reference to the dialysates in the middle of every sampling interval. At 480 min (8 h), a second and similar cefuroxime dose was administered. The sampling interval in the last 8 h was identical to that of the first 8 h. Dialysates were stored at −80 °C until analysis. Venous blood samples were centrifuged at 3000× *g* for 10 min and plasma was stored at −80 °C until analysis.

### 4.4. Microdialysis

Microdialysis is a catheter-based method for continuous sampling of the free unbound and active fraction of antibiotics in the interstitial space of the tissue of interest [43,44,45]. A semipermeable membrane at the tip of the catheter allows solutes to diffuse along the concentration gradient, but without achieving equilibrium, as the microdialysis catheter is constantly perfused. The concentrations obtained in the dialysates represent only a fraction of the actual concentration in the tissue. This fraction is referred to as the relative recovery and must be determined to obtain absolute tissue concentrations [46]. Relative recovery can be calculated during multiple calibration methods. In this study, meropenem was used as an internal calibrator [38].

Relative recovery:

$$Relative\;recovery\;\left(\%\right)=100\cdot \left(1-\frac{C_{dialysate}}{C_{perfusate}}\right)$$

$C_{dialysate}$ and $C_{perfusate}$ is the meropenem concentration in the dialysate and perfusate, respectivly.

Total tissue concentrations of cefuroxime ($C_{tissue}$) by correcting for relative recovery:

$$C_{tissue}=\frac{C_{dialysate}}{Relative\;recovery}$$

$C_{dialysate}$ is the cefuroxime concentration in the dialysate.

The microdialysis catheters (M Dialysis AB, Stockholm, Sweden), included CMA 63 catheters with a membrane length of 30 and 10 mm, and a 20 kD cut-off. TCMA 107 precision pumps generated a flow rate of 2 µL/min.

As microdialysis must be introduced into drill holes in bone, concerns have been raised whether microdialysis sampling of antibiotics actually reflects bone concentrations, a mixture of concentrations originating from the bone and the adjacent tissues or a blood clot filling the dead space around the probe. Three separate and independent studies have investigated these matters, indicating that the measured concentrations in drill holes in bone do reflect the actual bone concentrations [47,48,49]. Moreover, sampling from drill holes seems to reflect the true orthopaedic peri- and postoperative conditions. Therefore, microdialysis currently appears to be the most suitable method to obtain antibiotic bone concentrations; however, it is appreciated that no gold standard exists to validate these findings.

### 4.5. Liquid Chromatography Tandem Mass Spectrometry (LC-MS/MS)

The free unbound concentrations of cefuroxime in plasma and dialysates and meropenem in dialysates were quantified using a validated LC-MS/MS assay [37]. For cefuroxime the inter-run imprecisions were 14.2% at 0.01 µg/mL, 9.6% at 0.05 µg/mL, 2.6% at 5.00 µg/mL and 3.9% at 10.00 µg/mL. The lower limit of quantification (LOQ) was 0.01 µg/mL. For meropenem the inter-run imprecisions were 16.6% at 0.05 µg/mL, 3.9% at 5 µg/mL) and 5.6% at 10 µg/mL). LOQ was 0.050 µg/mL.

### 4.6. Pharmacokinetic Analysis and Statistical Considerations

*f*T > MIC for each target tissue and for each pig were estimated by linear interpolation for the S. aureus ECOFF MIC of 4 µg/mL, using Microsoft Excel (v. 16.49, Microsoft corp. Redmont, WA, USA). The investigated tissues were compared using one-way analysis of variance (ANOVA) with subsequent pairwise comparison of the compartments and dosing intervals.

The following pharmacokinetic parameters were calculated; the area under the concentration−time curves (AUC) by the linear up-log down trapezoidal method; peak drug concentration (C_max_) as the maximum of all the recorded concentrations and T_max_ as the time to reach C_max_; half-life (T_½_) as ln(2)/λeq, where λeq is the constant of terminal elimination rate, estimated by linear regression of the log concentration on time; tissue penetration was as the ratio of tissue AUC to free plasma AUC (AUC_tissue_/AUC_plasma_). All parameters were estimated separately for each compartment, using non-compartmental analysis, and compared using ANOVA followed by pairwise comparison of the compartments and dosing intervals. Descriptive statistics and analyses were performed in STATA (v. 16, StataCorpLLC, College Station, TX, USA). A correction for degrees of freedom by the Kenward–Roger approximation method was used due to the small sample size. The model assumptions were tested by visual assessment of residuals, fitted values and estimates of random effects. A *p*-value < 0.05 was accepted as statistically significant.

## 5. Conclusions

*f*T > MIC (4 μg/mL) and elimination from the induced orthopaedic deadspace was longer in comparison to plasma and bone following weight-adjusted cefuroxime dosing. Our results suggest a deadspace consolidation of a haematoma and a longer diffusion distance, resulting in a low cefuroxime turn-over, is why repeated cefuroxime administration at wound closure may improve deadspace *f*T > MIC (4 μg/mL). The role of adequate prophylactic antibiotic administration may be particularly important to protect the prosthesis from colonisation in a poorly perfused and potentially immunocompromised deadspace. Based on *S. aureus* theoretical targets, cefuroxime appears to be an appropriate prophylactic drug for the prevention of PJI. Validation of these findings in a clinical setting is warranted.

## Figures and Tables

**Table 1:** Comparison of weight groups regarding humeral head size, deadspace *f*T > MIC and AUC_deadspace_/AUC_plasma_.

Comparison of Weight groups	Group 1 53–57 kg	Group 2 73–77 kg	Group 3 93–97 kg
Humeral head size: Volume, mm^3^ (95% CI)	14,413 (10,941–17,885)	17,971 (14,499–21,443)	22,361 (18,889–25,833)
Deadspace *f*T > MIC, min (95% CI): First dosing interval	333 (241–424)	255 (163–346)	245 (148–341)
Deadspace *f*T > MIC, min (95% CI): Second dosing interval	321 (230–412)	378 (287–469)	315 (219–412)
AUC_deadspace_/AUC_plasma_ (95% CI): First dosing interval	1.87 (1.38–2.35)	1.77 (1.29–2.25)	1.27 (0.79–1.76)
AUC_deadspace_/AUC_plasma_ (95% CI): Second dosing interval	1.13 (0.65–1.61)	1.70 (1.22–2.19)	1.19 (0.68–1.71)

Humeral head size, mean volume in mm^3^ of the excised humeral head. *f*T > MIC, mean time above minimal inhibitory concentration of 4 μg/mL for *S. aureus.* AUC_deadspace_/AUC_plasma_, mean deadspace/plasma AUC-ratio (penetration).

**Table 2:** A pooled comparison across weight groups of mean *f*T > MIC for the two dosing intervals for plasma, deadspace and bone.

*f*T > MIC	First Dosing Interval	Second Dosing Interval	Total	*p*-Values 1st and 2nd Dosing Interval	*p*-Values Compartments
Plasma fT > MIC, min (95% CI)	124 (72–177)	160 (107–212)	284 (232–337)	0.206 *	<0.000 **
% fT > MIC (95% CI)	28 (16–39)	36 (24–47)	32 (26–37)		
Deadspace fT > MIC, min (95% CI)	277 (224–331)	338 (284–391)	605 (552–658)	0.039 *	<0.000 ***
% fT > MIC (95% CI)	62 (50–74)	75 (63–87)	67 (61–73)		
Bone fT > MIC, min (95% CI)	130 (77–182)	205 (152–257)	334 (282–387)	0.008 *	0.076 ****
*% fT > MIC (95% CI)*	*29 (17–40)*	*56 (34–57)*	*37 (31–43)*		

*f*T > MIC, mean time above minimal inhibitory concentration of 4 μg/mL for. %*f*T > MIC, mean % time above minimal inhibitory concentration of 4 μg/mL. * Comparison of first and second dosing interval within the compartment. ** Comparison of deadspace and plasma. *** Comparison of deadspace and bone. **** Comparison of bone and plasma.

**Table 3:** A pooled comparison across weight groups of mean time to reach a concentration of 4 μg/mL for the two dosing intervals for plasma, deadspace and bone.

TT4 μg/mL	First Dosing Interval	Second Dosing Interval	*p*-Values
Plasma: TT4, min (SD)	1 (0)	1 (0)	0.995 *
Deadspace: TT4, min (SD)	8 (8)	21 (23)	0.003 *
Bone: TT4, min (SD)	10 (16)	9 (9)	0.777 *

TT4 μg/mL, mean time in min to reach a concentration of 4 μg/mL. * Comparison of first and second dosing interval within the compartment.

**Table 4:** A pooled comparison across weight groups of key pharmacokinetic parameters for the two dosing intervals in plasma, deadspace and bone.

Parameter	First Dosing Interval	Second Dosing Interval	*p*-Values 1st vs. 2nd Dosing Interval
Plasma AUC, min µg/mL (95% CI)	2521 (1843–3201)	2868 (2190–3547)	0.404
Deadspace AUC, min µg/mL (95% CI)	4147 (3468–4826) ^ab^	3787 (3092–4482) ^ab^	0.394
Bone AUC, min µg/mL (95% CI)	1886 (1207–2565)	2621 (1942–3300)	0.079
Plasma C_max_, µg/mL (95% CI)	53 (49–58)	54 (49–59)	0.890
Deadspace C_max_, µg/mL (95% CI)	27 (22–32) ^ab^	19 (14–24) ^a^	0.011
Bone C_max_, µg/mL (95% CI)	18 (14–23)	18 (14–23)	0.976
Plasma T_max_, min (SD)	15 (0)	15 (0)	1.000
Deadspace T_max_, min (SD)	57 (29) ^a^	89 (48) ^ab^	<0.000
Bone T_max_, min (SD)	48 (14)	50 (15)	0.830
Plasma T_½_, min (SD)	46 (3)	97 (50)	0.338
Deadspace T_½_, min (SD)	151 (117)	407 (455) ^ab^	<0.000
Bone T_½_, min (SD)	74 (30)	149 (103)	0.169
Deadspace AUC_deadspace_/AUC_plasma_ (95% CI)	1.64 (1.36–1.92) ^b^	1.34 (1.05–1.62) ^b^	0.062
Bone AUC_bone_/AUC_plasma_ (95% CI)	0.75 (0.48– 1.03)	0.90 (0.62–1.18)	0.347

AUC, mean area under the concentration–time curve. C_max_, mean peak drug concentration. T_max_, mean time to C_max_. T_½_, half-life. AUC_tissue_/AUC_plasma_, mean tissue/plasma AUC-ratio (tissue penetration). ^a^ *p* < 0.05 when compared with plasma. ^b^ *p* < 0.05 when compared with bone.

## Data Availability

Data available on request from the authors.
